# Genome Sequence of an Extensively Drug-Resistant Strain of Klebsiella pneumoniae, Strain YN-1, with Carbapenem Resistance

**DOI:** 10.1128/genomeA.01279-14

**Published:** 2015-01-08

**Authors:** Hong-Li Tan, Yong Wang, Xue-Qin Cheng, Yan-Mei Huang, Wei Liu, Li-Juan Zhang

**Affiliations:** a Department of Laboratory Medicine, the Third People’s Hospital of Yunnan Province, Kunming, People’s Republic of China; b The National Institute for Communicable Disease Control and Prevention, China CDC, Beijing, People’s Republic of China; c College of Animal Science & Technology, Shihezi University, Shihezi, Xinjiang Province, People’s Republic of China

## Abstract

The emergence and spread of multidrug-resistant (MDR) Klebsiella pneumoniae has been regarded as one of the major challenges among health care-associated infections worldwide. Here, we report the draft genome sequence of an extensively drug-resistant (XDR) K. pneumoniae strain isolated in 2013 from Yunnan Province, China.

## GENOME ANNOUNCEMENT

Various resistance mechanisms have facilitated the emergence and spread of the MDR pathogen K. pneumoniae. Infections caused by MDR K. pneumoniae are associated with significant morbidity and mortality (1, 2). In this study, the extensively drug-resistant (XDR) K. pneumoniae strain was isolated from a bacteremia patient at a tertiary-care hospital in Kunming, the capital city of Yunnan Province, China. The strain exhibited resistance to imipenem and the broad-spectrum cephalosporins, as well as ciprofloxacin, levofloxacin, gentamicin, tobramycin, kanamycin, and furadantin (3). This XDR K. pneumoniae strain belongs to the multilocus strain type (MLST) ST29 and exhibited the CTX-M-15, SHV-1b-b, and TEM-1 genotypes. In addition, the strain carried IS*Ecp1* and IS*CR1*, which are gene-capturing elements (3).

The genome of XDR K. pneumoniae was sequenced using the Illumina HiSeq 2000 sequencing platform and generated 3,573,620 paired-end reads (approximately 56-fold coverage). The clean reads were assembled using the SOAP*denovo* assembler (http://soap.genomics.org.cn/) (4). Eighty-four contigs were obtained and connected to form 72 scaffolds based on the paired-end relationships of the reads. The *N*_50_ of the contigs was 285,951 bp, and the length of the largest contig was 433,951 bp. With regard to the scaffolds, the *N*_50_ was 420,159 bp, and the length of the largest scaffold was 1,306,066 bp. The order of the scaffolds was determined by length.

The draft genome of XDR K. pneumoniae is composed of 5,716,134 bp, with a G+C content of approximately 57.03%. The protein-coding regions were predicted by Glimmer (version 3.02 [http://www.cbcb.umd.edu/software/glimmer/]). In total, 5,562 coding genes were identified, for a total length of 4,980,171 bp and 87.12% coverage of the genome. Eighty-nine tRNA genes and 6 rRNA genes have putative functions assigned on the basis of the annotation. In addition, 19 gene islands and 6 prophages were detected in this chromosome by using SIGI-HMM (version 3.8) and the PHAge Search Tool (http://phast.wishartlab.com), respectively. Several resistance-associated genes were identified, including TEM, SHV, CTX, OXA-23, *qnrD*, *qnrS*, *gyrA*, *aadA1*, and 3 related gene cassettes of class I integrons. A comparative analysis of the genome from our isolate with published K. pneumoniae genomes will be reported in the future.

### Nucleotide sequence accession numbers.

The draft genome sequence of the XDR K. pneumoniae strain has been deposited in NCBI GenBank under the accession no. JRGE00000000.
